# Identifying local mechanisms for tumor-derived immunosuppression: an integrated phenotypic screening approach

**DOI:** 10.1186/2051-1426-2-S3-P135

**Published:** 2014-11-06

**Authors:** David J Klinke

**Affiliations:** 1 West Virginia University, Morgantown, WV, USA

## 

Simply stated, clinical response to an immunotherapy is proportional to the product of three quantities: the total number of tumor-infiltrating lymphocytes (TILs), the proportion of tumor cells that can be recognized by these TILs, and the cytotoxic efficacy of TILs directed against tumor cells. Recent clinical responses to Ipilimumab illustrate that TILs recognize tumor cells and that modifying immune checkpoints can increase the total number of T lymphocytes. Yet until we identify which of the many putative or potentially undiscovered local biochemical mechanisms limit the cytotoxic efficacy of TILs, the subset of patients that respond to these immune checkpoint modulators will be limited, as is the current state for Ipilimumab. To address this problem, we have developed a phenotypic screening workflow to identify local mechanisms that malignant cells use to suppress anti-tumor immunity. Specifically we have focused on identifying tumor-derived cues that locally suppress lymphocyte response to Interleukin-12, a key link between innate and adaptive immunity. By integrating high content assays, proteomics, and in silico methods, we identified tumor-derived Wnt-inducible signaling protein-1 (WISP1) as a paracrine suppressor of T cell response to IL-12 in vitro [1,2]. In patients with invasive breast cancer, we found that WISP1 is up-regulated in essentially all tumor samples from patients with invasive breast cancer [3]. While a type 1 immune response was correlated with improved survival in these patients, we also found that the expression of WISP1 correlated with a shift in immune polarization from a type 1 to a type 2 response. This observation is consistent with WISP1 as a paracrine regulator of immune response to IL12. Overall, this example serves to illustrate how a quantitative systems approach can be used to inform therapeutic strategies that enhance anti-tumor immunity and overcome local mechanisms for immunosuppression.
